# Parenting Styles and Adolescents’ School Adjustment: Investigating the Mediating Role of Achievement Goals within the 2 × 2 Framework

**DOI:** 10.3389/fpsyg.2017.01809

**Published:** 2017-10-16

**Authors:** Shiyuan Xiang, Yan Liu, Lu Bai

**Affiliations:** Institute of Developmental Psychology, Beijing Normal University, Beijing, China

**Keywords:** autonomy support, psychological control, achievement goals, school adjustment, adolescent

## Abstract

This study examines the multiple mediating roles of achievement goals based on a 2 × 2 framework of the relationships between parenting styles and adolescents’ school adjustment. The study sample included 1061 Chinese adolescent students (50.4% girls) between the ages of 12 and 19, who completed questionnaires regarding parenting styles (parental autonomy support and psychological control), achievement goals (mastery approach, mastery avoidance, performance approach, and performance avoidance goals) and school adjustment variables (emotion, students’ life satisfaction, school self-esteem, problem behavior, academic achievement, and self-determination in school). A structural equation modeling (SEM) approach was used to test our hypotheses. The results indicated that parental autonomy support was associated with adolescents’ school adjustment in an adaptive manner, both directly and through its positive relationship with both mastery and performance approach goals; however, parental psychological control was associated with adolescents’ school adjustment in a maladaptive manner, both directly and through its positive relationship with both mastery and performance avoidance goals. In addition, the results indicated that mastery avoidance goals suppressed the relationship between parental autonomy support and adolescents’ school adjustment, and performance approach goals suppressed the relationship between this adjustment and parental psychological control. These findings extend the limited literature regarding the 2 × 2 framework of achievement goals and enable us to evidence the mediating and suppressing effects of achievement goals. This study highlights the importance of parenting in adolescents’ school adjustment through the cultivation of different achievement goals.

## Introduction

Achievement goals are crucial determinants of students’ academic performance and school adjustment. Prior studies have shown that achievement goals can help us understand how students’ social environments affect their academic motivation (Dinger et al., 2013), emotions (Putwain et al., 2013), well-being (Tian et al., 2017), and performance (Diaconu-Gherasim and Măirean, 2016). However, few studies have analyzed why students develop and adopt different achievement goals. Because parents play a prominent role in shaping adolescents’ development (Grolnick and Ryan, 1989), it is important to evaluate how parental behaviors influence adolescents in endorsing certain goals while discouraging them from adopting other goals. In the present study, we investigated the mediating role of achievement goals, which were defined using a 2 × 2 framework (Pintrich, 2000; Elliot and McGregor, 2001) for the relationship between parental behaviors and school adjustment.

### Achievement Goals: Conceptual Differences and Various Influences

The achievement goal theory is one of the most prominent theoretical perspectives that explains and predicts the direction and intensity of individuals’ behavior in school-related situations. Early studies distinguished between mastery goals, which focused on the development of competence and the attainment of task mastery, and performance goals, which focused on the demonstration of competence relative to others (Dweck and Leggett, 1988). When individuals adopt mastery goals, failure feedback may be construed as helpful information to develop their competence; however, when individuals adopt performance goals, failure feedback implies a lack of normative ability (Elliot, 2005). Many researchers have proposed that a mastery/performance goal dichotomy exists. According to this idea, mastery goals are associated with adaptive patterns that include better academic performance, less anxiety, less depression, and superior well-being (Dweck and Leggett, 1988; Luo et al., 2011; Tian et al., 2017). In contrast, performance goals are associated with maladaptive patterns such as a lack of interest (Dweck and Leggett, 1988). However, Elliot et al. (1997) proposed that it may not be productive to consider all performance goals as maladaptive or directly opposed to mastery goals. Therefore, they revised the dichotomous achievement framework to form a trichotomous framework that bifurcated the conventional performance goals into approach and avoidance orientations. Performance approach goals denote aiming for demonstrating normative competence and outperforming others; performance avoidance goals denote aiming for not being the worst or not appearing stupid relative to others. Researchers have reported inconsistent results regarding these two types of performance goals. Performance avoidance goals have been positively related to poor outcomes, including a lack of interest, low grades, high anxiety, and self-handicapping strategies (Yeo et al., 2009; Dinger et al., 2013; Luo et al., 2013). Conversely, performance approach goals can have a positive relationship with grades, use of learning strategies, subjective well-being, and positive emotions (Lau and Nie, 2008; Liem et al., 2008; Tian et al., 2017). Barron and Harackiewicz (2001) have proposed a multiple goal perspective that claims it might be most adaptive for individuals to use both mastery goals and performance approach goals to reap the benefits from both types.

More recently, the approach and avoidance distinction has been incorporated with regard to mastery and performance goals (Pintrich, 2000; Elliot and McGregor, 2001). Mastery approach goals entail making efforts to improve one’s knowledge and skills, and mastery avoidance goals entail striving to avoid losing one’s skills and abilities or letting one’s development stagnate. Individuals who endorse mastery avoidance goals are concerned with being wrong in reference to themselves or the task, and might be considered “perfectionists” who always desire to be correct (Pintrich, 2000; Elliot and McGregor, 2001). In some studies, researchers have measured mastery avoidance goals and found that they were positively related to maladaptive outcomes, including negative emotions, threats to help-seeking, and less intrinsic motivation and perceived competence (Chiang et al., 2011; Luo et al., 2013; Putwain et al., 2013). However, other researchers determined that these goals were not related to performance (Cury et al., 2006; Elliot and Murayama, 2008; Yeo et al., 2009), whereas Diaconu-Gherasim and Măirean (2016) found that they were positively related to academic achievement. Overall, however, based on the results of prior studies, mastery avoidance goals are generally expected to produce less desirable consequences than mastery approach goals.

### Parenting Styles and Achievement Goals

Parents are generally concerned with, and involved in, shaping their children’s development. From a self-determination perspective, parental autonomy support and psychological control are two important factors that affect adolescents’ autonomous motivation and adjustment (Ryan and Deci, 2000; Vansteenkiste et al., 2005; Roth et al., 2009). Parental autonomy support refers to parental behaviors that encourage a child’s independent problem solving and decision making and promote the child’s autonomous regulation by considering the child’s perspective and providing a rationale and intrinsic value demonstration (Ryan and Deci, 2000; Roth et al., 2009). Parental psychological control refers to parental behaviors that are intrusive and manipulative of their children’s thoughts, feelings, and behaviors; these behaviors promote children’s introjected regulation and coerce them into conforming to the parents’ expectations (Barber, 1996; Assor et al., 2004).

Although parental autonomy support and psychological control are important factors for understanding motivation and adjustment, studies have only recently examined the influence of these two parenting styles on students’ achievement goals, particularly using a 2 × 2 framework. Generally, positive parental behaviors, including parental involvement, authoritarianism, and autonomy support, were positively associated with mastery goals. In contrast, negative parental behaviors, including control and permissiveness, were positively related to performance goals (Gonzalez et al., 2002; Gurland and Grolnick, 2005; Duchesne and Ratelle, 2010). However, when a 2 × 2 framework of achievement goals is used, the evidence for the links between parenting styles and achievement goals, particularly performance approach and mastery avoidance goals, is mixed. Specifically, some researchers have found that parental involvement, autonomy support, and control are positively related to performance approach goals (Kim et al., 2010; Luo et al., 2013). In contrast, other researchers have reported negative associations between performance approach goals and maternal involvement (Duchesne and Ratelle, 2010) or reported that a link does not exist between performance approach goals and parental autonomy support (Diaconu-Gherasim and Măirean, 2016). Because parental psychological control refers to a series of intrusive behaviors, including guilt-induction, contingent love, and instilling anxiety (Barber, 1996), adolescents who perceive psychological control might endorse performance approach goals to earn the approval of their parents and to reduce negative emotions. Conversely, because behaviors are instigated or directed by a positive and desirable event or possibility in approach motivation (Elliot and Covington, 2001), parental autonomy support might also contribute to an increase in approach motivation, including mastery and performance approach goals.

Few studies have examined the associations between parenting styles and mastery avoidance goals, and the authors of these studies have also reported mixed results. Luo et al. (2013) first examined the influence of parenting styles on mastery avoidance and found that parental control was positively associated with mastery avoidance goals and that parental involvement was not related to mastery avoidance goals. However, Diaconu-Gherasim and Măirean (2016) reported that parental rejection was negatively associated with mastery avoidance goals, but parental autonomy was positively associated with mastery avoidance goals. Gong et al. (2016) provided additional indirect evidence regarding the influence of parenting styles on mastery avoidance goals and reported positive associations between parental autonomy support and perfectionistic strivings, in addition to positive associations between parental psychological control and perfectionistic strivings and concerns. Because individuals who endorse mastery avoidance goals may be perfectionists and strive to avoid making mistakes (Pintrich, 2000; Elliot and McGregor, 2001), Gong et al.’s (2016) study provides additional indirect evidence for the assumption that both parental autonomy support and psychological control may positively predict adolescents’ mastery avoidance goals.

### The Mediating Role of Achievement Goals

Recent research has mainly focused on autonomous motivation as a potential mechanism underlying the role of autonomy-supportive and controlling parenting in children’s adjustment (Soenens and Vansteenkiste, 2005). Very few studies have addressed this issue by evaluating the role of achievement goals to explain how different styles of parenting influence children’s school adjustment. Certain studies have determined that parents’ and teachers’ emphases on mastery and performance goals predicted children’s personal goal orientation, which subsequently predicted children’s efficacy beliefs, coping strategies and behavioral and emotional engagement regarding their learning (Friedel et al., 2007; Gonida et al., 2009). Boon (2007) found that mastery goals mediated the relationships between parenting styles (parental warmth and strictness) and adolescents’ learning outcomes (self-efficacy and academic achievement). However, many of these studies only focused on mastery and performance orientations, and to our knowledge, only Luo et al. (2013) and Diaconu-Gherasim and Măirean (2016) examined the mediating role of achievement goals using a 2 × 2 framework for the relationship between parenting behaviors and adolescents’ adjustment. Luo et al. (2013) determined that parental involvement was associated with adaptive learning outcomes, including low anxiety, high perceived competence, and high achievement, partially or primarily because of its positive relationship with mastery approach goals. They also found that parental control was associated with maladaptive learning outcomes, such as low persistence, high anxiety, and low achievement, partially through its positive relationship with mastery and performance avoidance goals. Nevertheless, Diaconu-Gherasim and Măirean (2016) reported mixed results regarding the mediating role of achievement goals, particularly mastery avoidance ones. In this study, parental rejection negatively predicted academic achievement, mainly through its negative relationship with mastery approach and avoidance goals. They also found that parental autonomy positively predicted academic achievement, primarily because of its positive relationship with mastery avoidance goals. Generally, the mastery approach goal act as a positive mediator and the performance avoidance goal act as a negative mediator. However, the mediating roles of mastery avoidance and performance approach goals are unclear and additional studies are needed to examine the mediating role of these goals as regards the relationship between parenting styles and adolescents’ adjustment.

## The Current Study

The current study explored the mediating role of achievement goals based on a 2 × 2 framework of the relationship between parenting styles and school adjustment on a sample of adolescents. The first goal of the study was to investigate the relationship between achievement goals and school adjustment. Specifically, we hypothesized that mastery and performance approach goals would be associated with adaptive school adjustment and that mastery and performance avoidance goals would be associated with maladaptive school adjustment. The second goal was to investigate the relationship between parenting styles and achievement goals. We hypothesized that parental autonomy support would be positively associated with mastery approach, mastery avoidance, and performance approach goals and that parental psychological control would be positively associated with performance avoidance, performance approach, and mastery avoidance goals. The third goal was to explore whether achievement goals mediate the associations between parenting styles and adolescents’ school adjustment. We expected that the mastery approach and performance approach goals would assume mediating roles in the relationship between parental autonomy support and adolescents’ school adjustment. In addition, we expected that mastery avoidance and performance avoidance goals would assume mediating roles in the relationship between parental psychological control and adolescents’ school adjustment.

## Materials and Methods

### Participants

Participants were 1016 Chinese adolescents (50.4% girls) ranging from 12 to 19 years of age, with a mean of 14.81 (*SD* = 1.78). Data were collected from students in Wuhan and Urumqi, which were two typical provincial capital cities of central and western China respectively. Several schools received our request to carry out our study and three public schools (one in Wuhan and two in Urumqi) agreed to take part. The sample comprised 893 students who reported Han nationality, 113 who reported minority nationalities, and 8 who did not report their nationality. Most participants were in their first year of middle (33.2%) or high school (32.9%). Few families earned less than ¥3,000CNY (8.8%) or more than ¥20,000CNY (5.4%) per month while most families earned between ¥3,000CNY and ¥12,000CNY (74.5%). Most parents of the participants had obtained a high school or university degree (35.0 and 37.6% respectively).

### Measures

We adopted widely used standardized measures in this study. Validated Chinese versions of the measures were used when available. Measures not previously validated with Chinese samples (i.e., the Achievement Goal Questionnaire, the Student’s Life Satisfaction Scale, and the school self-esteem subscale of the Hare Self-Esteem Scale) were translated using the following procedure. The first author and the corresponding author, who are both researchers in the field of adolescent development and fluent in both English and Chinese, translated the measures from English to Chinese separately. Translations were compared and discrepancies were resolved among all three researchers to agree upon a common version. Then, three undergraduate students who major in Psychology and a junior high school psychology teacher checked the clarity of each of the questionnaire items. Final modifications were made by the three authors together. We report the psychometric properties of these three scales in the relevant following sub-sections.

#### Parental Autonomy Support

Parental autonomy support was assessed using Cheung and Pomerantz’s (2011) 12-item measure. The items in this scale were adopted from McPartland and Epstein (1977), Steinberg et al. (1992), and Robbins (1994). Participants were asked to indicate the extent to which their parents used autonomy-supportive practice on a 7-point scale (1 = not at all true, 7 = very true). A sample item is “My parents allow me to make choices whenever possible.” The mean of the 12 items was calculated, and higher numbers indicated greater parental support for autonomy. The measure has been successfully used in a Chinese sample before (Cheung and Pomerantz, 2011), and the Cronbach’s alpha was 0.92 in the current study.

#### Parental Psychological Control

Parental psychological control was assessed using Wang et al.’s (2007) 18-item measure. The items in this measure were adopted from Barber (1996) and Silk et al. (2003) or created by Wang et al. (2007). Participants were asked to indicate the extent to which their parents used psychologically controlling practice on a 7-point scale (1 = not at all true, 7 = very true). A sample item is “My parents tell me that I should feel guilty when I do not meet their expectations.” The mean of the eighteen items was calculated, and higher numbers indicated greater parental psychological control. The measure has been successfully used in Chinese samples before (Wang et al., 2007; Cheung and Pomerantz, 2011), and the Cronbach’s alpha was 0.91 in the current study.

#### Achievement Goals

The Achievement Goal Questionnaire (AGQ; Elliot and McGregor, 2001) was used to measure four types of achievement goals using a 2 × 2 framework. The questionnaire is a 12-item scale that includes four subscales of three items each: mastery-approach goals (e.g., “I want to learn as much as possible from this class”), mastery-avoidance goals (e.g., “I worry that I may not learn all that I possibly could in this class”), performance-approach goals (e.g., “It is important for me to do better than other students”), and performance-avoidance goals (e.g., “I just want to avoid doing poorly in this class”). Participants indicated the extent to which they believed that each item was true on a 7-point scale that ranged from “not at all true” to “very true.” The construct validity of this Chinese version was tested using confirmatory factor analysis. Fit indices indicated an adequate model fit: χ^2^(45) = 259.56, CFI = 0.95, TLI = 0.93, RMSEA = 0.069, 90% CI [0.061,0.077], and SRMR = 0.039. Cronbach’s alpha internal reliability coefficients were 0.77 for performance approach, 0.70 for performance avoidance, 0.80 for mastery approach and 0.76 for mastery avoidance.

#### Problem Behavior

We used Wang et al.’s (2010) Problem Behavior Scale to measure participants’ problem behavior. This scale consists of 7 items (e.g., fighting, stealing, alcohol use) to which participants were asked to indicate how often they had engaged in each activity during the last 3 months on a 5-point scale, ranging from “never” to “five times or more.” As in a previous study (Bao et al., 2015), the responses were averaged across all the items, with higher scores representing greater problem behavior. In the current study, the Cronbach’s alpha internal reliability coefficient was 0.71 for the entire scale.

#### Students’ Life Satisfaction

The Students’ Life Satisfaction Scale (SLSS; Terry and Huebner, 1995) was used to assess participants’ life satisfaction. Participants indicated the truth of seven statements (e.g., “I feel good about what’s happening to me”) using a 7-point scale that ranged from “not at all true” to “very true.” A high score on the scale indicates greater life satisfaction. The construct validity of this Chinese version was tested using confirmatory factor analysis. Fit indices indicated a good model fit: χ^2^(10) = 47.89, CFI = 0.99, TLI = 0.97, RMSEA = 0.061, 90% CI [0.044,0.079], and SRMR = 0.029. The Cronbach’s alpha internal reliability coefficient was 0.75 for the entire scale.

#### School Self-esteem

Participants’ school self-esteem was assessed with the school self-esteem subscale of the Hare Self-Esteem Scale (HSES; Shoemaker, 1980). This 10-item measure uses a 7-point scale that ranges from “not at all true” to “very true.” Sample items include “School is harder for me than for most other people.” In the present study, one item (“My teachers expect too much of me”) was removed because of its negative factor loading after reverse scoring the relevant items. The construct validity of this Chinese version was tested using confirmatory factor analysis. Fit indices indicated an adequate model fit: χ^2^ (22) = 132.96, CFI = 0.94, TLI = 0.90, RMSEA = 0.071, 90% CI [0.059,0.082], and SRMR = 0.043. The Cronbach’s alpha internal reliability coefficient was 0.75 for the entire scale.

#### Positive and Negative Emotions

The Positive and Negative Affect Schedule (PANAS; Watson et al., 1988) was used to measure participants’ positive and negative emotions. This questionnaire includes 10 positive and 10 negative affect descriptors that are randomly distributed. Participants indicated how often they had experienced each mood state during the past few weeks using a 7-point scale that ranged from “never” to “very often.” The scale has been successfully used in Chinese samples before (Liu et al., 2010), and the Cronbach’s alpha was 0.88 for positive affect and 0.86 for negative affect in the current study.

#### Academic Achievement

Because of the students’ right to confidentiality, we were unable to obtain their specific test scores from schools; therefore, we employed the self-report method for our data collection. Participants were asked to consider their grades in examinations for the most important subjects (Chinese, mathematics, English, physics, etc.) at the end of the last semester and report their academic achievement in school using a 6-point scale that ranged from “the top five percent” to “the last twenty percent.” The distribution of the academic achievement score fitted the normal distribution. Thus, scores were binned into a continuous variable as follows: The top 5 or 5–20% = 5; 20–40% = 4; 40–60% = 3; 60–80% = 2; the last 20% = 1. Higher numbers reflect better academic achievement.

#### Self-determination in School

We used a short 16-item version of the Self-Regulation Questionnaire - Academic (SRQ-A; Ryan and Connell, 1989; Van Ryzin et al., 2009) to assess students’ reasons for completing homework and trying to do well in school. Participants answered each item using a 7-point scale that ranged from “not at all true” to “very true.” The reasons for engaging in academic work reflect four different forms of regulation: External regulation is based on external pressures and reward, introjected regulation is based on internal pressure such as a feeling of guilt and anxiety, identified regulation is based on perceived value and worth, and intrinsic motivation is based on interest and inherent enjoyment. The Chinese version has been validated and widely used with Chinese samples (Zhang et al., 2011; Luo et al., 2014). In the current study, Cronbach’s alpha internal reliability coefficients were 0.72 for external regulation, 0.65 for introjected regulation, 0.60 for identified regulation and 0.84 for intrinsic motivation. The item scores from each of the four scales were averaged, then weighted according to their relationships with autonomy and summed to create the Self-Determination Index (SDI; Levesque et al., 2004). Consistent with previous research (Chirkov et al., 2003; Levesque et al., 2004), SDI was calculated as follows: SDI = (2 × Intrinsic) + (Identified) – (Introjected) – (2 × External). A higher score for SDI indicates a higher level of autonomy in school.

#### Controlling Variables

Data were collected on gender, age, and family socioeconomic status. To measure family socioeconomic status (Chen and Paterson, 2006), adolescents were presented with a drawing of a 10-rung ladder and asked to place their family on the ladder in comparison to other families.

### Procedure

This study was approved by the Ethics Committee of the School of Psychology, Beijing Normal University. Because the protocol was judged to pose a low risk and the data were collected and processed anonymously, letters that described the study and consent forms were only sent to school administrators and teachers, and oral consent was recommended and obtained from participants after a complete description of the study and before the data collection. Participants were told that they could omit any questions they felt uncomfortable answering and were free to withdraw from the study at any time during data collection. The set of questionnaires was completed during a 30-min session. Trained native research staff were available during completion of the questionnaire in case the participants had any questions. The teacher in charge of the class was also available to help with class discipline. Participants provided their own responses using the various rating scales and received a small gift (e.g., a highlighter) as a token of appreciation at the end of the session.

### Analysis

All inferential analyses were performed using Mplus 7 software. The rate for individuals omitting items was 1.29% for all considered items. For the initial descriptive analyses and correlations, SPSS version 20 was used, and missing data were addressed using a listwise deletion procedure. For other analyses, missing data were addressed using the Full Information Maximum Likelihood (FIML) approach implemented in Mplus. We used the Maximum Likelihood Estimator with Robust Standard Errors (MLR), which is appropriate for data that do not meet the assumption of multivariate normality (Kelloway, 2014). We used a three-step procedure to test our hypotheses. First, because item parceling is used to increase the stability of parameter estimates and is recommended if the relationships among latent variables are of focal interest (Little et al., 2002), each construct that was assessed using more than three items (with the exceptions of academic achievement and self-determination in school) was randomly aggregated into three item parcels. These served as manifest indicators of the respective latent variable. Second, confirmatory factor analysis (CFA) using Mplus 7 was performed to test the factorial structure of each scale. Third, we used a two-step procedure to test our hypotheses. First, we examined the associations between main study variables. Then, we estimated and evaluated the measurement model. If the result indicated a well-fitted measurement model, then the hypothesized outcomes of parental autonomy support and psychological control were examined separately. We analyzed the fit of all models using multiple indicators: The Chi square (χ^2^) with its associated degrees of freedom, the root mean-square error of approximation (RMSEA), the comparative fit index (CFI), the Tucker-Lewis index (TLI), and standardized root mean square residual (SRMR). For the CFI and TLI indices, values greater than 0.90 indicate an adequate fit to the data and values greater than 0.95 are considered excellent. For RMSEA and SRMR indices, values less than 0.08 are considered acceptable and values less than 0.06 reflect a good fit (Hu and Bentler, 1999).

## Results

### Descriptive Statistics

The relationships among all study variables, including the three covariates (i.e., gender, age, and family socioeconomic status), were presented in **Table 1**. Gender and achievement goals were significantly and negatively related, which indicated that girls tended to be more mastery-oriented than boys. Age was positively related to mastery approach and avoidance goals and negatively related to performance avoidance goals. Family socioeconomic status was positively associated with autonomy support and negatively associated with psychological control. It was also positively associated with mastery approach and performance approach goals. In addition, gender, age, and family socioeconomic status were all related to various school adjustment variables. Therefore, the three covariates were controlled for in the analysis.

**Table 1:** Descriptive statistics and correlations for the observed variables.

	1	2	3	4	5	6	7	8	9	10	11	12	13
(1) Autonomy support													
(2) Psychological control	-0.40**												
(3) Performance approach goals	0.12**	0.10**											
(4) Performance avoidance goals	0.00	0.23**	0.54**										
(5) Mastery approach goals	0.32**	-0.07*	0.47**	0.30**									
(6) Mastery avoidance goals	0.13**	0.12**	0.46**	0.50**	0.55**								
(7) Positive emotion	0.31**	-0.15**	0.14**	-0.10**	0.24**	-0.02							
(8) Negative emotion	-0.29 **	0.29**	0.10**	0.23**	-0.09**	0.18**	-0.28**						
(9) Students’ life satisfaction	0.39**	-0.29**	0.00	-0.14**	0.15**	-0.09**	0.40**	-0.41**					
(10) Problem behavior	-0.13**	0.15**	-0.09**	-0.00	-0.17**	-0.07*	-0.10**	0.14**	-0.13**				
(11) School self-esteem	0.39**	-0.30**	0.11**	-0.13**	0.32**	-0.08*	0.48**	-0.41**	0.50**	-0.20**			
(12) Academic achievement	0.21**	-0.14**	0.17**	-0.01	0.18**	0.02	0.17**	-0.16**	0.14**	-0.15**	0.39**		
(13) Self-determination in school	0.28**	-0.21**	0.05	-0.13**	0.20**	-0.02	0.44**	-0.28**	0.32**	-0.09**	0.37**	0.10**	
*Control variables*													
Gender	-0.07*	0.12**	-0.05	-0.01	-0.16**	-0.07*	0.05	-0.01	-0.09**	0.19**	-0.13**	-0.03	-0.03
Age	0.14**	-0.17**	-0.02	-0.09**	0.09**	0.07*	-0.09**	-0.02	0.01	-0.04	0.05	0.06	-0.28**
Family socioeconomic status	0.18**	-0.15**	0.06*	-0.08*	0.07*	-0.06	0.17**	-0.13**	0.24**	-0.08*	0.24**	0.10**	0.06
*M*	4.90	3.61	4.77	4.40	5.73	4.99	4.89	3.64	4.28	1.10	4.68	3.55	0.48
*SD*	1.23	1.17	1.42	1.43	1.26	1.40	1.08	1.19	1.04	0.30	0.91	1.26	5.23
Skew	-0.58	0.30	-0.35	-0.20	-1.20	-0.56	-0.36	0.12	-0.31	5.37	-0.07	-0.53	0.20
Kurt	0.17	-0.36	-0.39	-0.39	1.29	-0.17	-0.02	-0.50	0.15	42.36	-0.21	-0.74	-0.21


*Gender was dummy coded. ^∗^*p* < 0.05; ^∗∗^*p* < 0.01.*

### Associations between the Main Study Variables

First, we examined the relationship between parenting styles and school adjustment separately. The model of autonomy support had an adequate fit to the data: χ^2^(183) = 521.06, CFI = 0.95, TLI = 0.94, RMSEA = 0.043, 90% CI [0.039,0.048], and SRMR = 0.045. Results indicated that autonomy support was related to more positive emotions (β = 0.35, *SE* = 0.04, *p* < 0.001, *R*^2^ = 0.17), fewer negative emotions (β = -0.34, *SE* = 0.04, *p* < 0.001, *R*^2^ = 0.12), higher life satisfaction (β = 0.38, *SE* = 0.04, *p* < 0.001, *R*^2^ = 0.19), higher school self-esteem (β = 0.44, *SE* = 0.04, *p* < 0.001, *R*^2^ = 0.25), less problem behavior (β = -0.15, *SE* = 0.07, *p* < 0.05, *R*^2^ = 0.05), higher academic achievement (β = 0.20, *SE* = 0.03, *p* < 0.001, *R*^2^ = 0.04), and higher self-determination in school (β = 0.34, *SE* = 0.03, *p* < 0.001, *R*^2^ = 0.22). The model of psychological control also had an adequate fit to the data: χ^2^(183) = 511.88, CFI = 0.95, TLI = 0.94, RMSEA = 0.043, 90% CI [0.038,0.047], and SRMR = 0.044. Results indicated that psychological control was related to fewer positive emotions (β = -0.21, *SE* = 0.04, *p* < 0.001, *R*^2^ = 0.10), more negative emotions (β = 0.32, *SE* = 0.04, *p* < 0.001, *R*^2^ = 0.12), lower life satisfaction (β = -0.31, *SE* = 0.03, *p* < 0.001, *R*^2^ = 0.14), lower school self-esteem (β = -0.36, *SE* = 0.04, *p* < 0.001, *R*^2^ = 0.19), more problem behavior (β = 0.17, *SE* = 0.04, *p* < 0.001, *R*^2^ = 0.06), lower academic achievement (β = -0.15, *SE* = 0.03, *p* < 0.001, *R*^2^ = 0.03), and lower self-determination in school (β = -0.27, *SE* = 0.03, *p* < 0.001, *R*^2^ = 0.18).

Second, we examined the relationship between achievement goals and school adjustment to test our initial hypothesis. Fit indices indicated an adequate model fit: χ^2^(390) = 1147.35, CFI = 0.92, TLI = 0.90, RMSEA = 0.044, 90% CI [0.042,0.047], and SRMR = 0.047. Results indicated that mastery approach goals were associated with all seven school adjustment variables in an adaptive manner, such as more positive emotions (β = 0.44, *SE* = 0.10, *p* < 0.001), fewer negative emotions (β = -0.46, *SE* = 0.10, *p* < 0.001), higher life satisfaction (β = 0.42, *SE* = 0.08, *p* < 0.001), higher school self-esteem (β = 0.83, *SE* = 0.11, *p* < 0.001), less problem behavior (β = -0.31, *SE* = 0.10, *p* < 0.01), higher academic achievement (β = 0.24, *SE* = 0.07, *p* < 0.01), and higher self-determination in school (β = 0.43, *SE* = 0.08, *p* < 0.001). Mastery avoidance goals were associated with fewer positive emotions (β = -0.29, *SE* = 0.11, *p* < 0.01), more negative emotions (β = 0.42, *SE* = 0.13, *p* < 0.01), lower life satisfaction (β = -0.28, *SE* = 0.10, *p* < 0.01), lower school self-esteem (β = -0.71, *SE* = 0.13, *p* < 0.001) and lower academic achievement (β = -0.22, *SE* = 0.09, *p* < 0.05). Performance approach goals were positively associated with positive emotions (β = 0.30, *SE* = 0.10, *p* < 0.01) and academic achievement (β = 0.27, *SE* = 0.10, *p* < 0.01). Performance avoidance goals were negatively associated with positive emotions (β = -0.32, *SE* = 0.11, *p* < 0.01) and self-determination in school (β = -0.39, *SE* = 0.11, *p* < 0.001).

Third, we examined the relationship between parenting styles and achievement goals separately to test our second hypothesis. The model of autonomy support had an adequate fit to the data: χ^2^(113) = 467.11, CFI = 0.93, TLI = 0.91, RMSEA = 0.057, 90% CI [0.051,0.062], and SRMR = 0.051. Results indicated that autonomy support was positively related to mastery approach (β = 0.34, *SE* = 0.04, *p* < 0.001), mastery avoidance (β = 0.15, *SE* = 0.04, *p* < 0.01), and performance approach goals (β = 0.12, *SE* = 0.04, *p* < 0.01). In addition, the model of psychological control had an adequate fit to the data: χ^2^(113) = 474.67, CFI = 0.93, TLI = 0.91, RMSEA = 0.057, 90% CI [0.052,0.062], and SRMR = 0.052. Results indicated that psychological control was positively related to mastery avoidance (β = 0.16, *SE* = 0.04, *p* < 0.001), performance approach (β = 0.16, *SE* = 0.04, *p* < 0.001) and performance avoidance goals (β = 0.26, *SE* = 0.04, *p* < 0.001).

### Effects Mediated by Achievement Goals

The third goal of the study was to explore the mediating role of achievement goals between parenting styles and school adjustment. The measurement model was first tested and it provided an acceptable fit to the data: χ^2^(440) = 1100.52, CFI = 0.95, TLI = 0.94, RMSEA = 0.038, 90% CI [0.036,0.041], and SRMR = 0.043. All item parcels showed statistically significant loadings for the latent constructs, with all βs ≥ 0.54, and all *p*s < 0.001.

**FIGURE 1:**
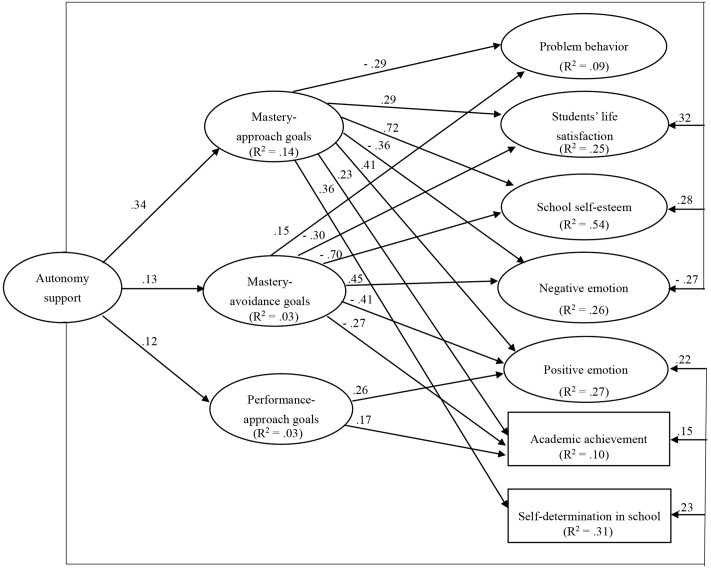
The multiple mediation model depicting the relations between parental autonomy support, multiple achievement goals, and school adjustment variables. Only significant path coefficients are reported. The values in the parentheses are percentage explained variances.

In the first structural model of autonomy support, three goals (mastery approach, mastery avoidance and performance approach goals) were entered simultaneously as mediators of the relationship between parental autonomy support and the seven school adjustment variables. This included all direct paths when the effects of gender, age, and family socioeconomic status on the three achievement goals and the seven school adjustment variables, and the effects of performance avoidance goals on school adjustment variables were controlled. Furthermore, as per previous research (Luo et al., 2013), the residuals of the four achievement goals were allowed to be correlated. This model had an adequate fit to the data: χ^2^(479) = 1281.35, CFI = 0.93, TLI = 0.92, RMSEA = 0.041, 90% CI [0.038,0.044], and SRMR = 0.044. **Figure 1** illustrated the significant paths in the resulting path model of parental autonomy support.

An examination of the specific indirect effects indicated that these were significant for parental autonomy support on school adjustment through achievement goals (see **Table 2**). Parental autonomy support was positively related to all seven school adjustment variables both directly and through its positive relationship with mastery approach, mastery avoidance and performance approach goals. Mastery approach and performance approach goals mediated the direct relationship between parental autonomy support and certain school adjustment variables because when these goals were accounted for, this direct relationship was diminished. Conversely, mastery avoidance goals suppressed the direct relationship between parental autonomy support and certain school adjustment variables because when these goals were accounted for, the direct relationship was enhanced.

**Table 2:** Standardized total, direct, and indirect effects through achievement goals.

Path	Total effects	Direct effects	Total indirect effects	Via mastery approach	Via mastery avoidance	Via performance approach	Via performance avoidance
Parental autonomy support							
To problem behavior	-0.15**	-0.07	-0.08**	-0.10*	0.02	-0.00	
To students’ life satisfaction	0.38**	0.32**	0.06**	0.10**	-0.03*	0.00	
To school self-esteem	0.45**	0.28**	0.17**	0.24**	-0.09**	0.01	
To negative emotion	-0.33**	-0.27**	-0.06*	-0.11**	0.06**	-0.00	
To positive emotion	0.33**	0.22**	0.11**	0.14**	-0.05*	0.03	
To academic achievement	0.21**	0.15**	0.06**	0.08**	-0.03*	0.02*	
To self-determination in school	0.35**	0.23**	0.12**	0.12**	-0.02	0.02	
Parental psychological control							
To problem behavior	0.16**	0.15**	0.01		0.02	-0.01	-0.00
To students’ life satisfaction	-0.28**	-0.23**	-0.05**		-0.05*	0.00	-0.00
To school self-esteem	-0.31**	-0.21**	-0.10**		-0.11**	0.02	-0.01
To negative emotion	0.30**	0.20**	0.10**		0.08*	-0.00	0.03
To positive emotion	-0.14**	-0.06	-0.08**		-0.04	0.04*	-0.07*
To academic achievement	-0.14**	-0.11**	-0.03		-0.03	0.05*	-0.05
To self-determination in school	-0.25**	-0.16**	-0.09**		-0.03	0.02	-0.08**


*^∗^*p* < 0.05; ^∗∗^*p* < 0.01.*

**FIGURE 2:**
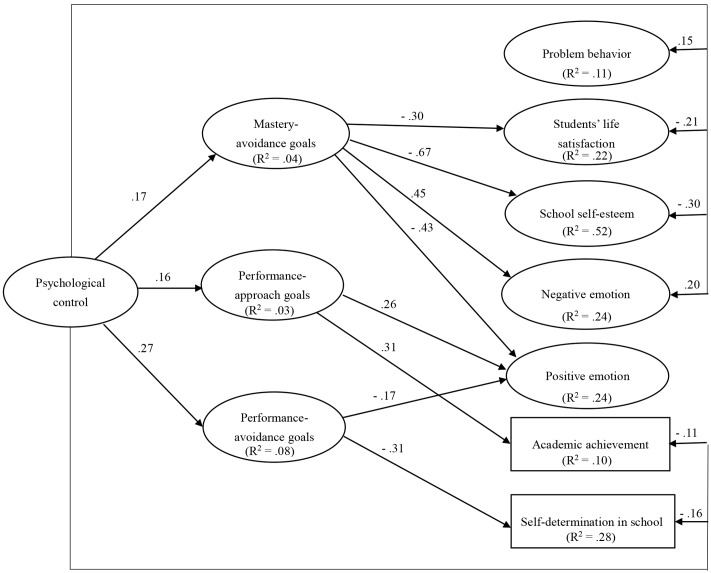
The multiple mediation model depicting the relations between parental psychological control, multiple achievement goals, and school adjustment variables. Only significant path coefficients are reported. The values in the parentheses are percentage explained variances.

In the second structural model, parental psychological control was the independent variable, and three goals (mastery avoidance, performance approach, and performance avoidance goals) were entered simultaneously as mediators. The effects of mastery approach goals on school adjustment variables were controlled, and other paths remained the same. This model also had an adequate fit to the data: χ^2^(474) = 1244.90, CFI = 0.93, TLI = 0.92, RMSEA = 0.040, 90% CI [0.037,0.043], and SRMR = 0.043. **Figure 2** illustrated the significant paths in the resulting path model of parental psychological control.

An examination of the specific indirect effects indicated that there were significant indirect effects of parental psychological control on school adjustment through achievement goals (see **Table 2**). In contrast to autonomy support, parental psychological control was negatively related to all the school adjustment variables and the relationships were partially mediated by mastery avoidance, performance approach, and avoidance goals. Notably, mastery avoidance and performance avoidance goals acted as mediators and performance approach goals acted as a suppressor between parental psychological control and certain school adjustment variables.

## Discussion

This study was designed to enhance our understanding of achievement goals based on a 2 × 2 framework. Specifically, we examined the important roles of achievement goals in the relationship between parenting styles and adolescents’ school adjustment. Three important results were obtained. First, mastery approach and performance approach goals were associated with school adjustment variables in an adaptive manner, but mastery avoidance and performance avoidance were associated with school adjustment variables in a maladaptive manner. Second, parental autonomy support was positively related to mastery approach, mastery avoidance, and performance approach goals and parental psychological control was positively related to mastery avoidance, performance approach, and performance avoidance goals. Third, mastery approach and performance approach goals mediated the relationship between parental autonomy support and adolescents’ school adjustment and mastery avoidance and performance avoidance goals mediated the relationship between parental psychological control and adolescents’ school adjustment. Furthermore, mastery avoidance goals suppressed the relationship between parental autonomy support and school adjustment, and performance approach goals suppressed the relationship between parental psychological control and school adjustment.

### Achievement Goals and School Adjustment

The results of this study support the hypothesis that mastery approach and performance approach goals are positively associated with adolescents’ adjustment, which is consistent with findings from previous research (Barron and Harackiewicz, 2001; Liem et al., 2008; Tian et al., 2017). Because performance approach goals may contribute to positive emotions and academic achievement even when the effect of mastery approach goals is controlled, our results confirmed the multiple goals perspective (Elliot et al., 1997; Barron and Harackiewicz, 2001) that setting both mastery approach and performance approach goals benefits students the most. Furthermore, we also found that mastery avoidance and performance avoidance goals harmed the adolescents’ adjustment, which was in agreement with previous research (Chiang et al., 2011; Luo et al., 2013; Dinger et al., 2013).

Additionally, it is important to note that mastery goals had a greater impact on adolescents’ adjustment when compared with performance goals. Previous research has suggested that performance approach goals are likely to transform to performance avoidance goals when students are faced with difficulties or the likelihood of failure (Luo et al., 2011). Because performance goals might change for different situations and because mastery goals are more stable, mastery approach goals were associated with more school adjustment variables in an adaptive manner when compared with performance approach goals, and mastery avoidance goals harmed more school adjustment variables than performance avoidance goals.

### Parenting and Achievement Goals

Our results indicated that adolescents with a higher family socioeconomic status reported a higher level of perceived autonomy support and a lower level of perceived psychological control. This is in line with previous research (September et al., 2016) that indicated that parents with high socioeconomic status were more authoritative and less harsh in their parenting. This highlights that it is important to provide opportunities for parents of low socioeconomic status to acquire skills that could enhance autonomy-supportive parenting.

Consistent with previous studies, parental autonomy support was positively related to mastery approach, mastery avoidance and performance approach goals (Gurland and Grolnick, 2005; Duchesne and Ratelle, 2010). Nevertheless, parental autonomy support was unrelated to performance avoidance goals, which contradicts prior studies that have reported a negative or positive association between these constructs (Gurland and Grolnick, 2005; Luo et al., 2013). These inconsistent results may be explained by considering how we measured parenting compared with previous research. For example, Gurland and Grolnick (2005) asked mothers to report their attitudes on autonomy support. In our study, the adolescents reported perceptions of parental autonomy support. In addition, our results were consistent with previous findings that parental psychological control was positively associated with mastery avoidance, performance approach and performance avoidance goals and unrelated to mastery approach goals (Gonzalez et al., 2002; Luo et al., 2013).

In terms of the associations between parenting styles and achievement goals, our data indicated that both styles of parental involvement (parental autonomy support and psychological control) were positively associated with performance approach and mastery avoidance goals. Positive associations of both parenting styles with performance approach goals might explain why performance approach goals were beneficial to adolescents’ school adjustment-related variables such as well-being and academic achievement (Liem et al., 2008; Tian et al., 2017) but were also likely to transform into performance avoidance goals, which might harm the adolescents’ adjustment (Luo et al., 2011, 2013). Moreover, it was interesting to find positive associations between parental autonomy support and mastery avoidance goals. Because autonomy-supportive parents generally have a high-quality relationship with their children (Niemiec et al., 2006), parental autonomy support might increase adolescents’ introjected regulation or their greater willingness to comply with their parents’ rules and willingness to engage in behaviors to obtain the approval of others (Zhou et al., 2009; Vansteenkiste et al., 2014). Therefore, it is reasonable that children of autonomy-supportive parents may feel guilty when they do something wrong and strive to avoid making mistakes.

### Achievement Goals: Mediators or Suppressors?

As hypothesized, mastery and performance approach goals mediated the positive impact of parental autonomy support on adolescents’ school adjustment, and mastery and performance avoidance goals mediated the negative impact of parental psychological control on adolescents’ school adjustment. Specifically, parental autonomy support was related to high academic achievement, both directly and through the two approach goals. In addition, parental autonomy support was associated with other school adjustment variables (less problem behavior, higher life satisfaction, higher school self-esteem, more positive emotion, less negative emotion, and higher self-determination in school) in an adaptive manner both directly and through mastery approach goals. Parental psychological control was associated with lower self-determination in school both directly and through performance avoidance goals and it was associated with other school adjustment variables (lower life satisfaction, lower school self-esteem, and more negative emotions) in a maladaptive manner both directly and through mastery avoidance goals. Generally, parenting styles were related to adolescents’ school adjustment primarily through mastery approach and avoidance goals; however, performance goals played a role in explaining the associations between parenting and adolescents’ adjustment.

In Luo et al.’s (2013) study, parenting style was measured as the involvement in and control of students’ learning, and adolescents’ adjustment was measured as self-engagement in learning activities, persistence, achievement, and anxiety in math class. In Diaconu-Gherasim and Măirean’s (2016) study, the acceptance versus rejection dimension and the autonomy versus psychological control dimension were measured as parenting styles, and only academic achievement was examined. However, in our research, we focused on parental autonomy support and psychological control, which had great impacts on adolescents’ motivation. We also measured adolescents’ school adjustment in different dimensions including adolescents’ emotion, life satisfaction, self-esteem, problem behavior, academic achievement, and self-determination in school. In addition, both in Luo et al.’s (2013) and Diaconu-Gherasim and Măirean’s (2016) studies, they only found a mediating role of achievement goals between parenting styles and school adjustment, and their results were seemingly contradictory to each other. For example, Luo et al. (2013) found that mastery avoidance goals could not explain the relationship between parental involvement and adolescents’ adjustment, while Diaconu-Gherasim and Măirean (2016) found that mastery avoidance goals could be a mediator to explain the relationship between parental autonomy and adolescents’ academic achievement. Contrary to prior studies, utilization of the 2 × 2 framework enabled us to demonstrate a second intermediary role: Suppressor variables for achievement goals. Specifically, parental psychological control was associated with performance approach goals, and these goals counteracted the overall inimical influence of parental psychological control on positive emotions and academic achievement. Cury et al. (2006) reported a similar result in the social-cognitive domain; these authors reported that performance approach goals could suppress the negative effect of entity theory on academic performance. Because performance approach goals were likely to transform to performance avoidance goals when adolescents were faced with difficulties and pressure (Luo et al., 2011), the seemingly positive effect of psychological control through performance approach goals might exist only when adolescents are engaged in easy tasks and this positive effect cannot completely counteract the detrimental effect of parental psychological control. In addition, parental autonomy support was associated with mastery avoidance goals, and these goals suppressed the overall positive influence of parental autonomy support on adolescents’ school adjustment, including students’ life satisfaction, school self-esteem, positive emotion, less negative emotion, and academic achievement. Therefore, perceptions of parental autonomy support did not produce a uniformly positive effect on adolescents’ school adjustment, and perceptions of parental psychological control did not produce a uniformly negative effect. Our results suggest that each specific goal adopted has an important impact on the eventual school adjustment variables.

### Limitations and Future Research

Although this study makes several contributions, it also has some limitations that should be taken into account. First, this study was based on cross-sectional survey data, and the proposed causal sequence of parenting, achievement goals and school adjustment cannot be fully justified from this design. Although the postulated directions of arrows in the models are based on the achievement goal theory and previous research, adolescents’ school adjustment may affect their parents’ behavior, which could be tested using a longitudinal design in the future. Second, all data were based on adolescents’ self-reports; therefore, it would be beneficial to use multiple methods of assessment in future studies. Third, because of students’ right to confidentiality, schools were not allowed to provide the students’ exact scores, and the self-report method was used to measure students’ academic achievement. Finally, key individual difference factors could be explored in future studies to explain why the same parenting style predicts different achievement goals.

### Implications

Despite these limitations, our findings have possible implications related to the links among adolescents’ perceptions of parenting styles, achievement goals, and school adjustment. First, the results revealed that both approach goals were positively related to school adjustment and both avoidance goals were negatively related to this. Therefore, parents and teachers may consider promoting adolescents’ school adjustment through the cultivation of mastery approach and performance approach goals. Second, the results suggest that parenting plays an important role in shaping adolescents’ adjustment through its effect on adolescents’ endorsement of achievement goals. Therefore, significant parental autonomy support and low control would be beneficial during adolescence. Third, this study added to the limited literature regarding the use of a 2 × 2 achievement goal model, particularly regarding mastery avoidance goals and the mediating role of achievement goals between parenting and adolescents’ school adjustment. Our utilization of the 2 × 2 framework enabled us to provide evidence for the mediating and suppressing effects of achievement goals. Therefore, in addition to parenting style, the specific goal that is adopted also has a very important influence on the eventual school adjustment variables. Autonomy-supportive parents and controlling parents should all consider the specific goals that adolescents endorse to help create a better environment for their children.

## Ethics Statement

This study was carried out in accordance with the recommendations of “the Ethics Committee of School of Psychology, Beijing Normal University” with written informed consent from all subjects. All subjects gave written informed consent in accordance with the Declaration of Helsinki. The protocol was approved by the “the Ethics Committee of School of Psychology, Beijing Normal University.”

## Author Contributions

SX, YL, and LB substantially contributed to the conception and the design of the work. SX and LB contributed to the acquisition of the data. SX and YL analyzed and interpreted the data. SX prepared the draft and YL and LB reviewed it critically and gave important intellectual content. All authors approved the final version of the manuscript for submission.

## Conflict of Interest Statement

The authors declare that the research was conducted in the absence of any commercial or financial relationships that could be construed as a potential conflict of interest.
